# Artesunate acts through cytochrome c to inhibit growth of pediatric AML cells

**DOI:** 10.1038/s41598-023-49928-y

**Published:** 2023-12-16

**Authors:** Kristen S. Hill, Erin E. Schuler, Sally R. Ellingson, Jill M. Kolesar

**Affiliations:** 1 Markey Cancer Center, University of Kentucky, Lexington, KY USA; 2 Department of Pathology and Laboratory Medicine, University of Kentucky, Lexington, KY USA; 3 Division of Biomedical Informatics, UK College of Medicine, Cancer Research Informatics, University of Kentucky, Lexington, KY USA; 4 Department of Pharmacy Practice and Research, College of Pharmacy, University of Kentucky, Lexington, KY USA

**Keywords:** Cancer therapy, Haematological cancer, Leukaemia, Acute myeloid leukaemia, Cancer, Paediatric cancer, Cancer, Haematological cancer

## Abstract

Artesunate is a derivative of artemisinin, an active compound isolated from *Artemisia annua* which has been used in Traditional Chinese Medicine and to treat malaria worldwide. Artemisinin derivatives have exhibited anti-cancer activity against both solid tumors and leukemia. The direct target(s) of artesunate are controversial; although, heme-bound proteins in the mitochondria have been implicated. We utilized computational modeling to calculate the predicted binding score of artesunate with heme-bound mitochondrial proteins and identified cytochrome c as potential artesunate target. UV–visible spectroscopy showed changes in the absorbance spectrum, and thus protein structure, when cytochrome c was incubated with artesunate. Artesunate induces apoptosis, disrupts mitochondrial membrane potential, and is antagonized by methazolamide in pediatric AML cells indicating a probable mechanism of action involving cytochrome c. We utilized a multi-disciplinary approach to show that artesunate can interact with and is dependent on cytochrome c release to induce cell death in pediatric AML cell lines.

## Introduction

It is predicted that over 10,000 new cases of pediatric cancer will be diagnosed in the United States of America in 2022 and over 25% of pediatric cancers will be leukemias^1^. Most pediatric leukemia is classified as either acute lymphoblastic leukemia or acute myeloid leukemia (AML)^1^. AML is a hematological malignancy that arises from myeloid precursor cells. In the context of pediatric AML, the 5-year survival rate is 65–70% with approximately 30% of patients developing recurrent disease after standard of care chemotherapy, which includes cytarabine and daunorubicin^2^.

The prevalence of relapse in pediatric AML underscores the importance of identifying new therapies which can be used to treat recurrent disease. Recently several studies have reported that the anti-malarial drug artesunate exhibits anti-cancer activity against multiple solid tumors, such as colon, bladder, lung, and ovarian cancer^3–8^. Additionally, artesunate has also been shown to have anti-cancer activity in adult and pediatric AML cell lines^8–12^.

Several mechanisms of action have been implicated in artesunate's anti-cancer activity in both solid tumors and hematological malignancies, including, induction of reactive oxygen species (ROS), induction of apoptosis, G1 cell cycle arrest, DNA damage, and the inhibition of angiogenesis^3,4,8–10,13–16^. Many of the pathways linked to artesunate’s activity in both malaria and cancer converge on the induction of ROS and the mitochondria. A recent study suggests that the mechanism of action of artemisinin and derivatives in malaria treatment may be heme-activated promiscuous covalent binding to the parasite’s proteins when present in red blood cells^17^. Another study showed that artesunate targeted cancer stem cells by altering mitochondrial metabolism and ultimately inducing mitochondrial dysfunction^18^. Additionally, non-covalent binding of artesunate to mitochondrial targets is possible, as heme groups have been shown to cause conformational change in protein kinases that could potentially alter their druggable states and lead to heme-activated binding^19^.

Mitochondria are the location of oxidative phosphorylation and a major source of ROS production in cells as electrons are transferred between multiple complexes required to produce ATP. Many of the proteins involved in the electron transport chain within the mitochondria contain heme bound iron which is required for artesunate to increase ROS in malaria^14,17,20,21^. One such protein that has been linked to both ROS production and the induction of apoptosis is cytochrome c. Under normal cellular conditions cytochrome c shuttles between a reduced and oxidized states carrying electrons from complex III, cytochrome c reductase, to complex IV, cytochrome C oxidase^22–24^. In response to pro-apoptotic signals cytochrome c becomes uncoupled from this chain and is released into the cytoplasm where it regulates the formation and activity of the apoptosome, resulting in caspase activation^22–25^.

We utilized a multi-disciplinary approach to identify a potential direct target of artesunate. First, we used computational modeling to identify cytochrome c as a potential binding partner. Cytochrome c is of interest not only based on the favorable predicted binding score with artesunate but also due to its known activity in regulating apoptosis and ROS. Next, we used UV–visible spectroscopy to visualize the effect of artesunate on cytochrome c protein structure when incubated together in vitro. Lastly, we utilized pediatric AML cell lines to determine if artesunate’s anti-cancer effects were consistent with a mechanism of action involving regulation of cytochrome c.

## Results

### Identification of potential artesunate binding partners

**Figure 1:**
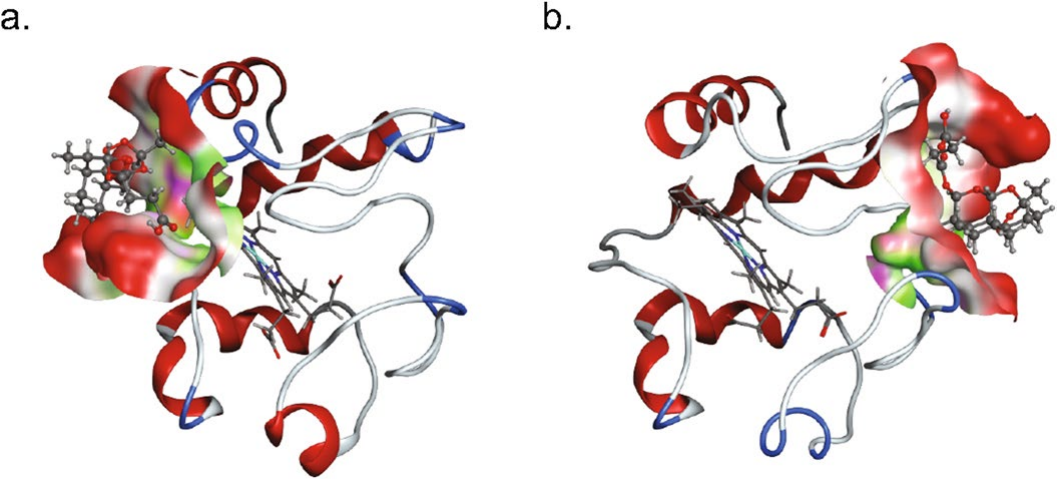
Artesunate in top ranked binding site of reduced (**a**) and oxidized (**b**) cytochrome c as determined by MOE. (**a**) Artesunate is predicted to bind near the heme group in reduced cytochrome c. (**b**) Artesunate is predicted to bind oxidized cytochrome c on the opposite side of the protein from the heme group. Surface map depicted near ligand with polar (magenta), hydrophobic (green), and exposed (red) surfaces colored.

Molecular Operating Environment software (MOE) was utilized to model docking of artesunate to a number of heme bound proteins in the mitochondria including cytochrome c, Supplemental Table 1. While not being the highest ranked predicted binding protein with artesunate, cytochrome c was selected for these studies due to its role in the regulation of apoptosis and ROS^22–25^. The docking of artesunate in the binding pocket with the highest propensity for binding artesunate for both reduced and oxidized cytochrome c is shown in Fig. 1. A surface map of the protein is displayed near artesunate. The proteins were superposed before docking and are displayed in the same orientation. Artesunate’s most likely binding site on reduced cytochrome c (Fig. 1a) is located in close proximity to the heme group, while artesunate’s strongest predicted binding site to oxidized cytochrome c (Fig. 1b) is on the opposite side of the protein away from the heme group. Supplemental Fig. 1 shows the location of important amino acid residues relative to the predicted binding location of artesunate in the reduced (S1a) and oxidized (S1b) structures. The structures have been independently rotated to best view the important residues in this figure.

### *Artesunate’s interaction with cytochrome c *in vitro

**Figure 2:**
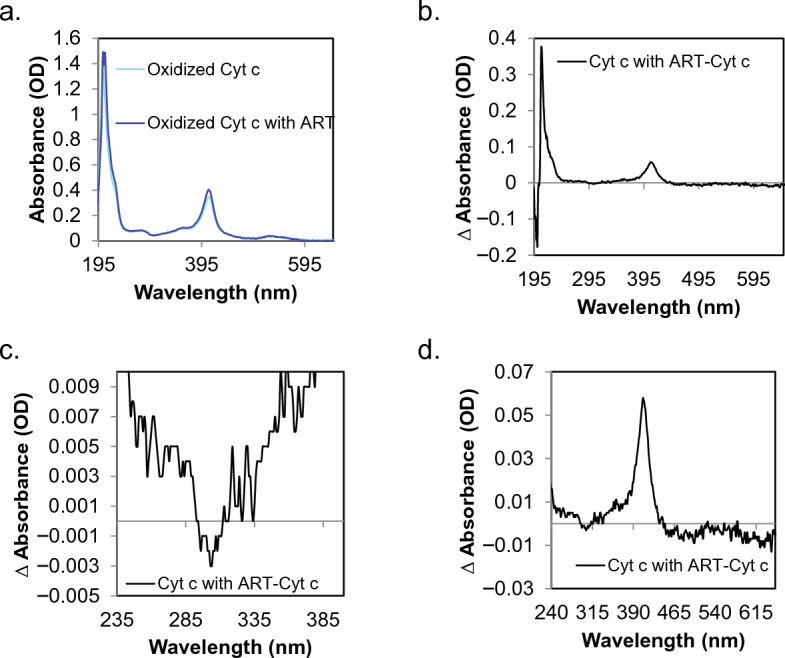
UV–visible spectroscopy show changes in oxidized cytochrome c when incubated with artesunate. (**a**) Full spectra of oxidized cytochrome c with (dark blue) and without (light blue) artesunate. (**b**) A difference spectrum between the two traces of oxidized cytochrome c with and without artesunate. In this difference spectra we can see a shift in the spectral region associated with the protein back bone (206–220 nm). (**c**) Zoomed in panel of the difference spectra for the aromatic residues reveals bleaching in the aromatic region which includes the absorbance of both tyrosine and tryptophan residues. (**d**) Zoomed in panel for the heme moiety shows an increase in relative intensity, but not a spectral shift, of the Soret band of the heme moiety.

**Figure 3:**
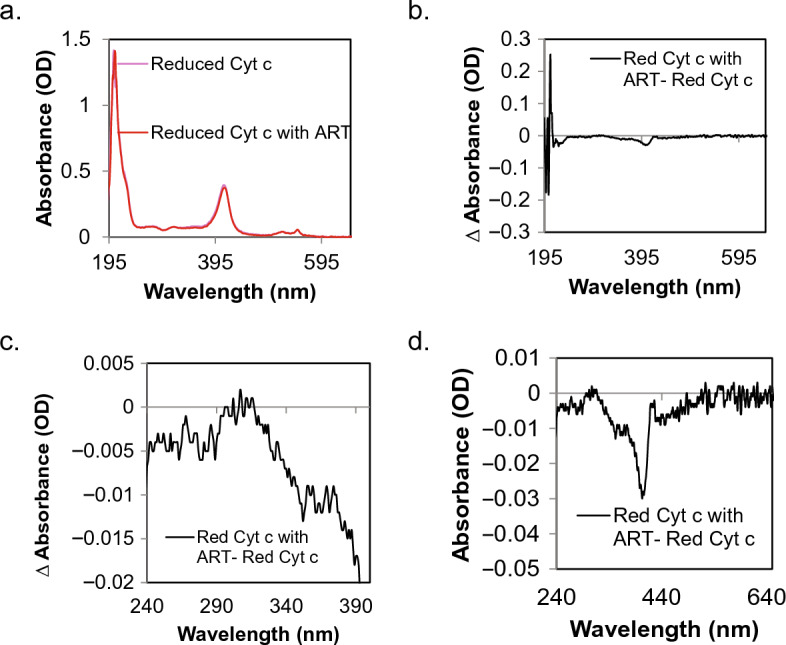
UV–visible spectroscopy show changes in reduced cytochrome c when incubated with artesunate. (**a**) Full spectra of reduced cytochrome c with (red) and without (pink) artesunate. (**b**) A difference spectrum between the two traces of reduced cytochrome c with and without artesunate. In this difference spectra we can see a shift in the spectral region associated with the protein back bone (206–220 nm). (**c**) Zoomed in panel of the difference spectra for the aromatic residues shows no change in the absorbance bands of the aromatic residues. (**d**) Zoomed in panel for the difference spectra highlighting the heme moiety shows a shift in the absorbance at the Soret band that this is unique to reduced cytochrome c.

We next used UV–visible spectroscopy to investigate the interaction of artesunate with cytochrome c in vitro; specifically, to quantify changes in the structure of both reduced and oxidized cytochrome c, as well as the heme moiety, when incubated with purified artesunate. The structure of oxidized cytochrome c was confirmed by the electronic transitions at 409 nm and 530 nm; while the structure of reduced cytochrome c was confirmed by electronic transitions at 415 nm, 520 nm and 550 nm. Both oxidized cytochrome c (Fig. 2a,b) and reduced cytochrome c (Fig. 3a,b) spectra revealed a red shifted absorbance in the transitions associated with the amide backbone of the protein structure between 206-220 nm when incubated with artesunate. Further evidence of artesunate binding cytochrome c is observed in the spectral region associated with the absorbance of aromatic residues in the region of 257–280 nm; where the oxidized structure in the presence of artesunate demonstrated a decrease in intensity relative to oxidized cytochrome in the absence of artesunate and is visible as a bleach in the difference spectrum of cytochrome c with artesunate subtracted from the neat protein spectrum, Fig. 2c. Absorbance bands associated with aromatic residues in the structure of reduced cytochrome c in the presence of artesunate revealed no significant change in intensity or spectral shift relative to the spectrum of the neat protein, Fig. 3c. With respect to the heme moiety, a shift in the maximum peak absorbance was observed in the reduced cytochrome c structure from 411 to 413 nm in the presence of artesunate, Fig. 3a. The difference spectrum between cytochrome c with and without artesunate in the reduced structure also revealed a bleach in the absorbance at 403 nm with a concomitant increase in intensity at 420 nm, Fig. 3d. No shift in the maximum absorbance peak was observed in the oxidized structure with both spectra revealing a peak absorbance of 409 nm and a mild increase in intensity with oxidized cytochrome c in the presence of artesunate, Fig. 2d. Together this data suggests that artesunate may have unique binding modalities between the oxidized and reduced forms, where the association of artesunate with reduced cytochrome c favors binding near the heme binding cleft.

### Artesunate treatment inhibits cytochrome c oxidase activity

**Figure 4:**
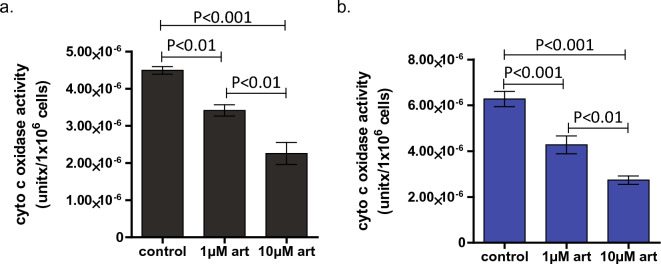
Dose dependent inhibition of cytochrome c oxidase activity by artesunate. Mitochondria were isolated from MV4-11 (**a**) and THP-1 (**b**) cells following treatment with vehicle control, 1 µM, or 10 µM artesunate for 24 h and the activity of cytochrome c oxidase from these mitochondria was assessed by a kinetic in vitro assay. Cytochrome c oxidase activity was assessed by determining the change in OD550nm overtime and normalizing this change to the cellular input during the mitochondrial isolation. The mean cytochrome c oxidase activity per 1 × 10^6^ cells was graphed + / − SD from three independent experiments. Statistical significance was determined by One-way ANOVA with a Newman-Keuls Multiple comparison test.

The predicted binding site of artesunate on reduced cytochrome c overlaps with the known interacting surface between reduced cytochrome c and complex IV, cytochrome c oxidase; therefore, we hypothesize that artesunate treatment could inhibit cytochrome c oxidase activity. To test this, we performed an in vitro cytochrome c oxidase activity assay using mitochondria isolated from artesunate treated MV4-11 and THP-1 cells. In this assay, reduced cytochrome c is mixed with the isolated mitochondria and the absorbance at 550 nm is measured every 30 s for 1 h. If cytochrome c is oxidized the absorbance at 550 nm decreases and the rate of this decrease over a set period of time is used as a measure of cytochrome c oxidase activity. Cytochrome c oxidase activity was normalized to the number of cells used in the mitochondrial isolation to account for any cell death induced by the 24 h artesunate treatment, Fig. 4a,b. Vehicle control treated MV4-11 cells had a mean cytochrome c oxidase activity of 4.49 × 10^–6^ + / − 2.02 × 10^–7^ units/1 × 10^6^ cells. MV4-11 cells treated with 1 µM artesunate treatment had a significantly reduced (*P* < 0.01, One-way ANOVA with Newman-Keuls Multiple comparison test) cytochrome c oxidase activity which was 3.42 × 10^–6^ + / − 3.74 × 10^–7^ units/1 × 10^6^ cells. This was further reduced to 2.26 × 10^–6^ + / − 7.25 × 10^–7^ units/1 × 10^6^ cells (*P* < 0.001 vs. control, *P* < 0.01 vs. 1 µM artesunate) by treatment with 10 µM artesunate, Fig. 4a. Vehicle control treated THP-1 cells had a slightly higher cytochrome c oxidase activity level at 6.28 × 10^–6^ + / − 8.10 × 10^–7^ compared to the MV4-11 cells. Artesunate treatment of the THP-1 cells also resulted in a dose dependent decrease in cytochrome c oxidase activity to 4.28 × 10^–6^ + / − 9.57 × 10^–7^ (*P* < 0.001 vs. control) with 1 µM artesunate and 2.74 × 10^–6^ + / − 4.52 × 10^–7^ (*P* < 0.001 vs. control, *P* < 0.01 vs. 1 µM) with 10 µM artesunate, Fig. 4b.

### Artesunate has anti-cancer activity in pediatric AML

**Figure 5:**
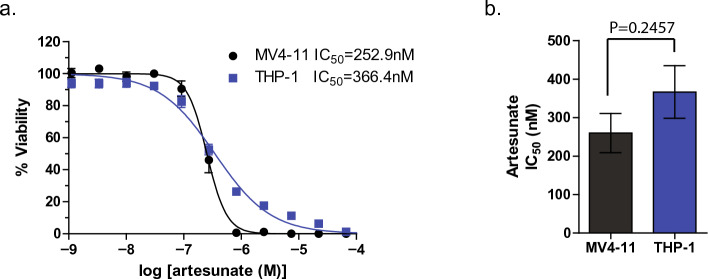
MV4-11 and THP-1 cells are equally sensitive to artesunate. (**a**) MV4-11 (black) and THP-1 (blue) cells were treated with increasing concentrations of artesunate for 96 h prior to assessing cell viability. Data is expressed as the % viability normalized to matched 0.1% DMSO control treated cells and is graphed as the mean + / − SD from 5 independent experiments. (**b**) IC_50_ values calculated from 5 independent experiments are graphed and an unpaired two-tailed t-test showed no significant difference (*P* = 0.2457) between the IC_50_ for artesunate between cell lines tested.

We first tested if artesunate has anti-cancer activity in pediatric AML cell lines by assessing the viability of cells after 96 h incubation with increasing concentrations of artesunate. MV4-11 cells had an IC50 for artesunate of 252.7 nM + / − 28.68 (mean + / − SD), while in THP-1 cells the IC50 was 348.1 nM + / − 57.54, Fig. 5a. When the IC50s from 5 independent experiments were compared using a two-tailed t-test there was not a statistically significant difference (*P* = 0.2457) between the IC50 for artesunate in MV4-11 and THP-1 cells, demonstrating that artesunate has activity in the mid-nanomolar range for both AML cell lines tested, Fig. 5b.

### Artesunate’s activity requires cytochrome c release from the mitochondria

**Figure 6:**
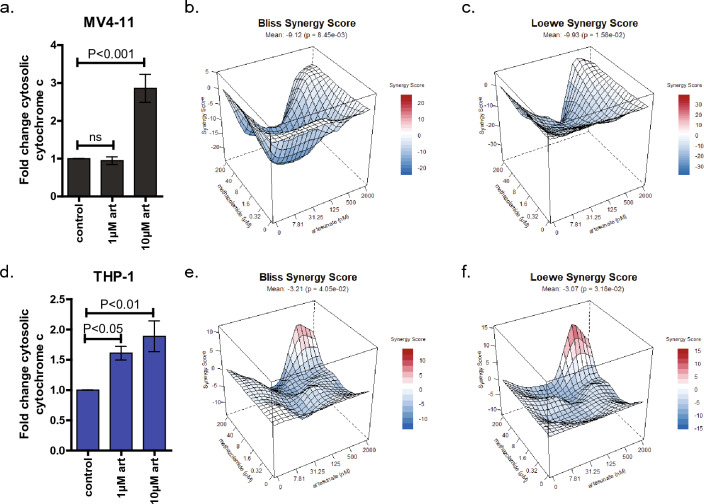
Artesunate induces cytochrome c release which is required for its anti-cancer activity. MV4-11 (**a**) and THP-1 (**d**) cells were treated with vehicle control, 1 µM or 10 µM artesunate for 24 h prior to cell fractionation and analysis of cytoplasmic cytochrome c by ELISA. Cytochrome c levels ng/µg total protein were determined by comparing the corrected absorbance at 540 nm to a known standard curve of purified human cytochrome c. The data was normalized to matched controls and is graphed as the mean fold change + / − SD from three independent experiments. Statistical significance was determined by One-way ANOVA with a Tukey’s multiple comparison test. (**b**–**c**, **e**–**f**)Cells were treated using a 6 × 6 grid layout with a combination of increasing concentrations of artesunate and methazolamide for 96 h prior to assessing cell viability. Viability data was normalized to matched 0.2% DMSO (vehicle) controls and analyzed using R synergyfinder package to calculate mean BLISS and Loewe synergy score for each cell line. BLISS synergy plot for MV4-11 (**b**) and THP-1 (**e**) with mean and p-value listed. Loewe synergy plot for MV4-11 (**c**) and THP-1 (**f**). Both synergy models tested show significant antagonism between artesunate and methazolamide in both cell lines.

The first step to investigate if interaction of artesunate and cytochrome c plays a role in artesunate’s anti-cancer activity was to determine if artesunate treatment results in cytochrome c release from the mitochondria. To do this we assessed the level of cytochrome c in the cytoplasm following artesunate treatment of MV4-11 and THP-1 cells. The cells were treated with artesunate for 24 h and both cytoplasmic and mitochondrial fractions were collected. The amount of cytochrome c in the cytoplasmic fractions was determined using a human cytochrome c ELISA and the results for each sample was normalized to the amount of total protein in each well. The levels of cytochrome c (ng/µg total protein) were compared to the matched vehicle control within each experiment and the mean fold change + / − SD from four independent experiments is shown in Fig. 6a,d. In MV4-11 cells treatment with 1 µM artesunate did not significantly change the amount of cytochrome c in the cytoplasm (fold change 0.946 + / − 0.383); however, 10 µM artesunate significantly increased cytoplasmic cytochrome c (fold change 2.859 + / − 1.381, *P* < 0.001), Fig. 6a. In THP-1 cells both 1 µM artesunate (fold change 1.610 + / − 0.357, *P* < 0.05) and 10 µM artesunate (fold change 1.887 + / − 0.803, *P* < 0.01) significantly increased the amount of cytochrome c in the cytoplasmic fraction, Fig. 6d.

To determine if cytochrome c release from the mitochondria was important to the anti-cancer activity of artesunate we next investigated what effect methazolamide, an inhibitor of cytochrome c release^26^, would have on pediatric AML cell lines when used alone or in combination with artesunate. Across all concentrations tested, methazolamide alone and no effect on the viability of MV4-11 or THP-1 cells, Supplemental Fig. 2. To assess the effect of methazolamide in combination with artesunate we utilized a 6 × 6 grid viability assay to assess drug interactions as being additive, synergistic, or antagonistic using two different methods, BLISS^27^ and Loewe^28,29^. In both cell lines the synergy scores for each model were negative which indicates an antagonistic relationship between the two drugs being tested, Fig. 6b–c,e–f. Specifically, in MV4-11 cells the mean (+ / − SD) BLISS synergy score was − 9.124 + / − 2.318 (*p* = 0.00845) and the mean Loewe synergy score was − 9.927 + / − 3.132 (*p* = 0.0158); while in THP-1 cells the mean BLISS score was − 3.210 + / − 1.201 (*p* = 0.0405) and the mean Loewe score was − 3.070 + / − 1.303 (*p* = 0.0318). The observations that artesunate induces cytochrome c release from the mitochondria and the strong antagonism between artesunate and methazolamide in both synergy models suggests that artesunate's anti-cancer activity requires cytochrome c release from the mitochondria.

### Artesunate disrupts mitochondrial membrane potential and induces apoptosis

**Figure 7:**
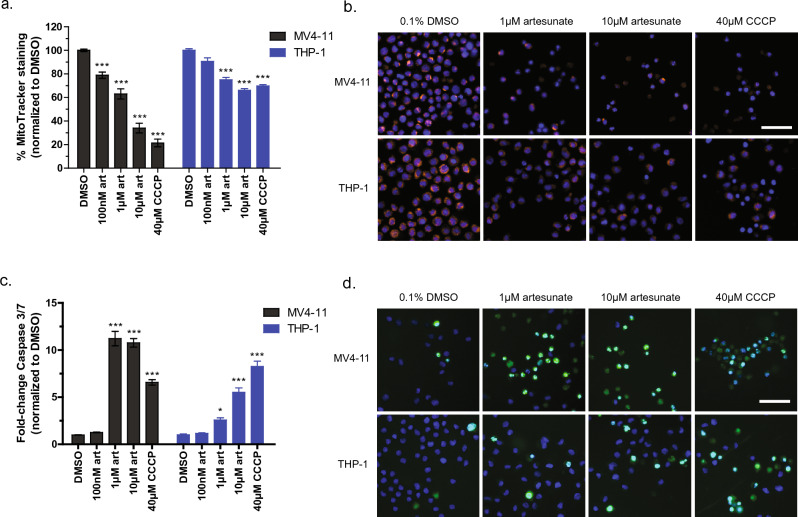
MitoTracker and Caspase 3/7 activity staining following treatment with increasing concentrations of artesunate for 48 h. (**a**) Quantification of the mean MitoTracker orange staining per cell normalized to matched 0.1% DMSO control treated cells and graphed as % MitoTracker staining + / − SD from 3 independent experiments. (***P < 0.001; ANOVA) (**b**) Representative images from one experiment. scale bar = 50 µm. (**c**) Quantification of the mean caspase 3/7 activity staining per cell normalized to matched 0.1% DMSO control treated cells and graphed as fold-change staining + / − SD from 3 independent experiments. (*P < 0.05, ***P < 0.001; ANOVA) (**d**) Representative images from one experiment. scale bar = 50 µm.

Cytochrome c release from the mitochondria is a key inducer of cellular apoptosis and is known to disrupt the mitochondrial membrane potential^22–25^. Based on the increased amount of cytochrome c in the cytoplasm with artesunate treatment and the antagonism between artesunate and methazolamide we hypothesized that artesunate could induce apoptosis and/or impair mitochondrial membrane potential in pediatric AML cell lines. To test this hypothesis, we treated MV4-11 and THP-1 cells with 0, 100 nM, 1 µM, or 10 µM artesunate for 48 h prior to staining the cells with MitoTracker orange, a dye that is used to label intact mitochondria, or with a caspase 3/7 activity stain. CCCP (carbonyl cyanide 3-chlorophenylhydrazone) is known to disrupt mitochondrial membrane potential by increasing membrane permeability to protons which is a trigger for apoptosis^30,31^; therefore, 40 µM CCCP was used as a positive control for these experiments. DMSO (vehicle) treated cells were used as a negative control for these experiments and data is expressed as the mean signal intensity of MitoTracker normalized to the cell line matched DMSO control. Results are expressed as a percentage of staining + / − SD with the DMSO control treated cells having 100% staining. In MV4-11 cells a significant decrease in staining with MitoTracker orange was observed after treatment with 100 nM (78.91% + / − 6.41), 1 µM (62.99% + / − 12.98), 10 µM artesunate (34.05% + / − 12.18), Fig. 7a. In THP-1 cells a significant decrease in staining with MitoTracker was observed following treatment with 1 µM (75.02% + / − 5.79) and 10 µM artesunate (66.14% + / − 4.24), Fig. 7a; while the effect of 100 nM artesunate was not significant at 90.70% + / − 7.25.

To determine if in addition to altering the mitochondrial membrane potential artesunate also induces apoptosis, cells were treated with increasing concentrations of artesunate for 48 h and then stained with CellEvent caspase-3/7 green detection reagent. This stain shows enhanced fluorescent signal when bound to DNA in the nucleus, but the unprocessed dye cannot enter the nucleus and thus is not detected in the green fluorescent channel. When caspase 3 or caspase 7 are enzymatically active they can proteolytically cleave the protein allowing the dye to enter the nucleus, bind to DNA, and the fluorescent signal can be detected; therefore, an increase in green fluorescent signal is a measure of increased caspase 3/7 activity and thus of apoptosis. Data is expressed as the fold change in caspase 3/7 activity staining when compared to matched vehicle (DMSO) treated cells. An 11.22-fold + / − 2.64 and 10.76-fold + / − 1.56 increase in caspase 3/7 activity was observed following treatment of MV4-11 cells with 1 µM or 10 µM artesunate, respectively, Fig. 7c. THP-1 cells exhibited a dose dependent increase in caspase 3/7 activity with 1 µM artesunate causing a 2.58-fold + / − 0.83 and 10 µM artesunate resulting in a 5.52-fold + / − 1.66, Fig. 7c, compared to DMSO treated cells. In both MV4-11 and THP-1 cells treatment with 100 nM artesunate did not significantly increase caspase 3/7 activity having a 1.26-fold + / − 0.08 and 1.16-fold + / − 0.17 increase in caspase 3/7 staining, respectively. This data supports that artesunate functions, at least in part, to disrupt the mitochondrial membrane and induce apoptosis by direct binding with cytochrome c and that artesunate's activity is dependent on the release of cytochrome c from the mitochondria.

## Discussion

### Structural implications of artesunate binding cytochrome c

Cytochrome c is a heme bound protein normally found in the intermembrane space of the mitochondria where it serves to shuttle electrons from complex III to complex IV as an integral member of the electron transport chain^22–24^. When cytochrome c becomes uncoupled from the electron transport chain it can leave the mitochondria and enter the cytoplasm where it, along with apoptotic protease activating factor 1 (Apaf-1), regulates the formation and function of the apoptosome and leads to the induction of apoptosis through caspase 9 activation^22–25^. Computational modeling of artesunate binding to cytochrome c revealed that artesunate’s predicted binding site differed for the reduced and oxidized forms of cytochrome c, Fig. 1. In the case of reduced cytochrome c, the highest scoring potential binding site was located near the heme group suggesting that artesunate could interact with the heme bound iron in reduced cytochrome c. The formation of an endoperoxide bridge between artesunate and heme bound iron is required for the anti-malarial activity of artesunate through the induction of ROS and ferroptosis^14,17,20,21^. Based on the predicted binding site of artesunate with reduced cytochrome c, a similar mechanism could, at least in part, be responsible for the anti-cancer activity of artesunate, but further studies are required to prove this hypothesis. UV–visible spectroscopy demonstrated shifts in the absorbance spectra associated with the amide backbone as well as in features associated with the heme moiety, specifically the Soret band of reduced cytochrome c in response to the addition of artesunate indicating that artesunate binding to reduced cytochrome c induces structural changes to the heme group, Fig. 3. The observed changes in reduced cytochrome c upon incubation with artesunate support that artesunate is binding to reduced cytochrome c near the site predicted by computational modeling. This potential binding site is also in close proximity (within 15 angstroms) of 6 key lysine residues (K8, 13, 72, 73, 86, and 87), Supplemental Fig. 1a, that are involved in reduced cytochrome c binding to cytochrome c oxidase in complex IV of the electron transport chain^32^. The close proximity of the predicted binding sites of artesunate to the known binding site of cytochrome c oxidase could result in interference with the ability of reduced cytochrome c to bind cytochrome c oxidase in the presence of artesunate. Further studies, including comparing the dissociation constant of artesunate and cytochrome c to that of cytochrome c and cytochrome c oxidase, are needed to draw a mechanistic conclusion. However, the fact that artesunate treatment inhibits cytochrome c oxidase activity, Fig. 4, serves to indirectly support this hypothesis.

The highest scoring predicted binding site for artesunate to oxidized cytochrome c is located away from the heme binding site and from the surface involved in binding to cytochrome c oxidase, Fig. 1b, but its location could still be functionally significant with regards to artesunate's anti-cancer activity. UV–visible spectroscopy reveals a significant bleaching of the absorbance spectrum associated with aromatic amino acids, such as tyrosine and tryptophan, in oxidized cytochrome c when incubated with artesunate, Fig. 3. This indicates that artesunate binds to oxidized cytochrome c in close proximity to key tryptophan or tyrosine residues. Both tyrosine 48 (Y48) and tryptophan 59 (W59) are located within 15 angstroms of the highest scoring predicted binding site for artesunate on oxidized cytochrome c, Supplemental Fig. 1b. Tyrosine 48 has been identified through proteomic analysis as well as experimentally to function in cytochrome c induced apoptosis^33,34^ . Specifically, phosphorylation of Y48 or mutation to glutamic acid (Y48E), which mimics tyrosine phosphorylation^35^, inhibits caspase activation by the apoptosome^34^. Given that artesunate induces apoptosis, we hypothesize that artesunate binding to oxidized cytochrome c could be a steric inhibitor of the phosphorylation of Y48, which would functionally promote cytochrome c dependent apoptosis. The predicted artesunate binding site is also located near W59 which is important because the nitrogen on the indole moiety interacts with the heme via hydrogen bonding and this amino acid acts to stabilize the hydrophobic core of cytochrome c^36^. If artesunate binding causes changes in the structure of cytochrome c near W59, which the UV–visible spectroscopy data indicates, then it could also destabilize the hydrogen bonds between W59 and the heme group resulting in the observed spectral shifts in features of the heme moiety, Fig. 2. In addition to the role W59 plays in stabilizing the heme group through hydrogen bonding it also plays a role in the stability of the hydrophobic core of the cytochrome c structure and artesunate binding could functionally destabilize the global structure of oxidized cytochrome c likely altering its function in electron transport^36^.

**Figure 8:**
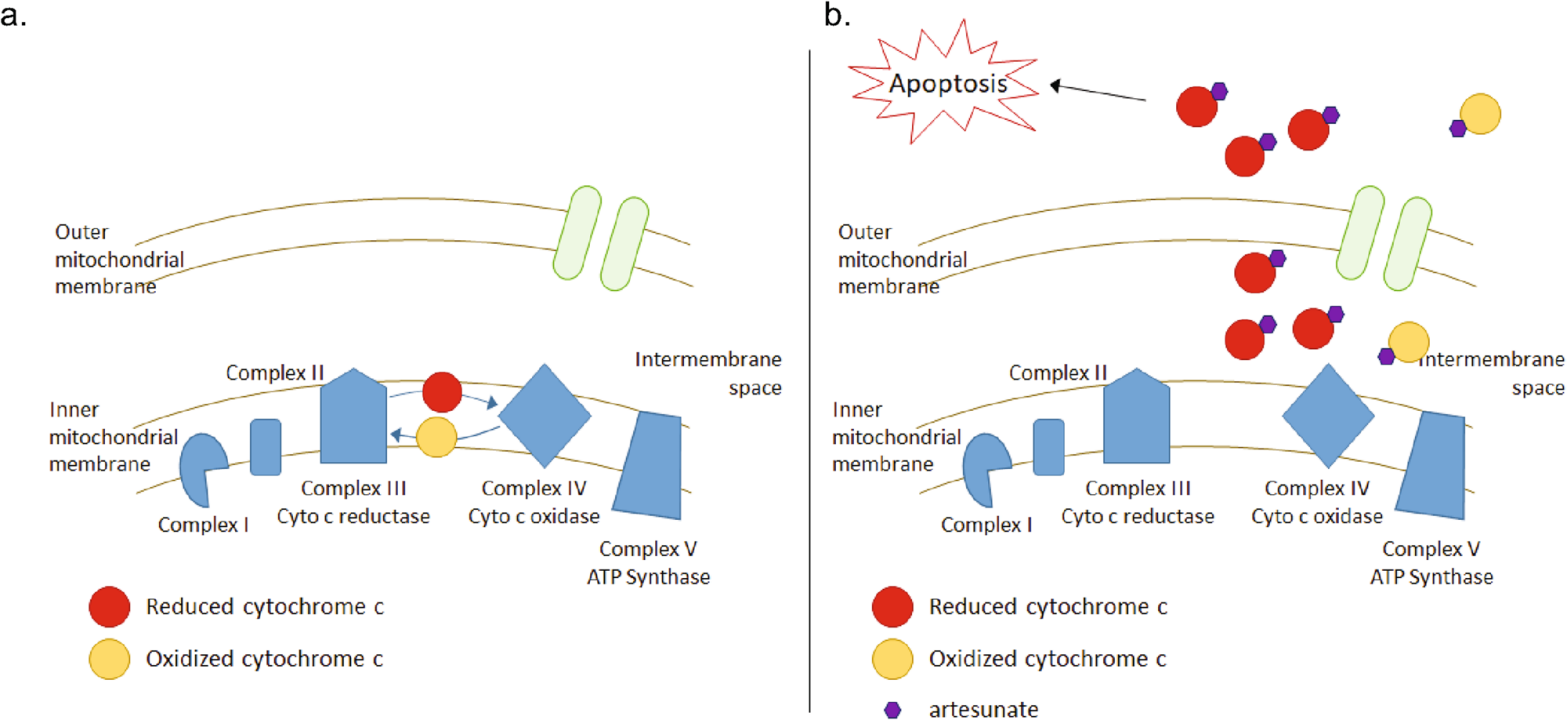
Scheme depicting hypothesized effect of artesunate binding to reduced and oxidized cytochrome c. (**a**) Prior to artesunate treatment heme-bound iron in cytochrome c serves as an electron shuttle between complex III and complex IV in the electron transport chain located in the inner mitochondrial membrane and this function requires cytochrome c to transition from a reduced (red) to an oxidized (yellow) state. (**b**) When artesunate (purple) is added it binds to both reduced and oxidized cytochrome c. When bound to reduced cytochrome c artesunate blocks the surface required for cytochrome c to interact with complex IV resulting in a buildup of reduced cytochrome c which is uncoupled from the electron transport chain. When bound to oxidized cytochrome c artesunate induces changes in the protein structure around tryptophan 59 and tyrosine 48 which could alter the stability of the heme group. These functions combine to induce cytochrome c release from the mitochondria and the induction of apoptosis by artesunate.

When the computational modeling and UV–visible spectroscopy data from this study is considered together it supports that artesunate can bind to cytochrome c regardless of the oxidization state of cytochrome c. Additionally, based on the predicted binding sites, artesunate binding to both reduced and oxidized cytochrome c could result in small conformational changes and/or steric inhibition of normal cytochrome c function leading to the uncoupling of cytochrome c from the electron transport chain and the release of cytochrome c from the mitochondria thus promoting cytochrome c’s apoptotic functions, Fig. 8.

### *Artesunate has anti-cancer activity in AML *via* cytochrome c*

It is well established in the literature that artesunate’s anti-malaria activity is linked to the induction of ROS within *Plasmodium falciparum* and increased cellular ROS is also a mechanism of action for artesunate in cancer^8,13–17,20,21^. What is not known is how artesunate induces ROS in cancer cells. We showed that not only does artesunate induces the release of cytochrome c from the mitochondria, but that artesunate’s anti-cancer activity is dependent on cytochrome c release. Specifically, the addition of methazolamide, which inhibits cytochrome c release, significantly antagonizes the anti-cancer activity of artesunate, Fig. 6. Cai et al^22^. showed that pharmacological inhibition of electron transport with cyanide or antimycin A, as well as cytochrome c release from the mitochondria, stimulated superoxide production by diverting electron flow from the middle portion of the electron transport chain. A similar mechanism to induce ROS could be activated by artesunate binding to cytochrome c which could functionally uncouple cytochrome c from the middle portion of the electron transport chain resulting in cytochrome c release and superoxide production.

A study by Gotsbacher et al^37^. identified Bcl-2 associated agonist of cell death (BAD) as a target of artesunate. Specifically, artesunate binding BAD prevented BAD phosphorylation at serine 136 and promoted the interaction of BAD with Bcl-_XL_ (B-cell lymphoma-extra large) which in turn promoted cytochrome c release and induced apoptosis^37^. These findings complement our data showing the importance of cytochrome c release for artesunate’s anti-cancer activity and demonstrate that the effects of artesunate on cytochrome c release could be two-fold. When cytochrome c is released into the cytoplasm it interacts with Apaf-1 to form the apoptosome and actives caspase-9 which in turn activates caspase-3 and caspase-7 resulting in cellular apoptosis^22–25^. It is important to note that the ability of cytochrome c to regulate apoptosis is independent of its oxidative state^22–25^. Therefore, artesunate could induce apoptosis when bound to either reduced or oxidized cytochrome c. Consistent with this in pediatric AML cells treatment with low micromolar concentrations of artesunate result in a significant increase in caspase-3/7 activity, Fig. 7c,d.

Additionally, the release of cytochrome c and the activation of caspase-3/7 can both result in the loss of the mitochondrial membrane potential which initiates further increases in cellular ROS and enhances cells apoptotic response^22^. The loss of mitochondrial membrane potential was quantified through staining with MitoTracker orange, and a significant loss of mitochondrial membrane potential was observed after treatment with artesunate in pediatric AML cell lines, Fig. 7a,b. It is important to note that the effects of artesunate on cell viability, mitochondrial membrane potential, and apoptosis all occurred at clinically relevant drug concentrations which are lower than the Cmax observed in clinical trials assessing artesunate in the treatment of solid tumors^38,39^.

### Strengths/limitations

This study is unique in that it utilized computational modeling to predict artesunate-protein interactions, UV–visible spectroscopy to demonstrate in vitro drug-protein interactions, and cell based phenotypic and drug synergy experiments to identify and support cytochrome c as a direct target of artesunate. The data from these three different fields supports that cytochrome c, regardless of its oxidative state, is a direct target of artesunate and that artesunate requires cytochrome c release from the mitochondria to exert its anti-cancer activity. One limitation of this study is that the MOE docking did not consider the heme and Vina does not consider the charge on metals if the heme which could be important to artesunate binding and activity, especially if artesunate’s activity in cancer cells is dependent on the formation of an endoperoxide bridge forming with the heme-bound iron like is seen in malaria^14,17,20,21^. However, we think this is unlikely, given that malaria infections occur in the heme rich environment of erythrocytes which lack mitochondria and cytochrome c, there is covalent binding of artesunate to dozens of plasmodium proteins causing inactivation, but no evidence of apoptosis. Our proposed anti-cancer mechanism of action differs substantially, where we demonstrate release of cytochrome c from cellular mitochondria which subsequently induces apoptosis. Furthermore, these calculations can be considered a prediction of how well artesunate is attracted to a binding pocket. Future studies will use more advanced methods to take the state of the heme into account in the binding energy calculations.

Additionally, we recognize that UV–visible spectroscopy is reporting on the global protein structure and is not capable of identifying the precise amino acids involved in artesunate binding. Further studies using fluorescent or infrared spectroscopy in combination with amino acid labeling are needed to elucidate the specific amino acids responsible for artesunate binding. Even with these limitations the strength of this study lies in the fact that the computational modeling, in vitro binding (UV–visible spectroscopy), and cell-based studies were all completed independently but when combined, the findings are consistent with cytochrome c being a direct target of artesunate and required for artesunate’s anti-cancer activity. This manuscript is also the first reporting that cytochrome c is a direct target of artesunate.

### Future directions

It is likely that given the array of mechanisms by which artesunate has been shown to exert its anti-cancer activity in solid tumors that additional direct binding partners may be involved in artesunate’s activity. For this study we focused on identifying potential non-covalent binding interactions between artesunate and heme bound proteins within the mitochondria. Some research suggests that artesunate may act through covalent binding in the presence of heme and additional tools will be utilized in future studies to probe these for other direct artesunate targets. Additionally, having identified cytochrome c as a direct target of artesunate, future studies will also focus on identifying rationale drug combinations, these could include combining artesunate with small molecules that promote cytochrome c release or inhibit proteins that function to restrict cytochrome c within the mitochondria.

### Conclusions

In this study we used computational modeling to identify the predicted binding sites between artesunate and mitochondrial heme proteins and identified both reduced and oxidized cytochrome c as potential direct targets of artesunate. Using UV–visible spectroscopy we showed that incubation of artesunate with both reduced and oxidized cytochrome c resulted in shifts in the absorbance spectrum of the protein which indicates not only artesunate binding to both states of the protein but supported the different predicted binding sites identified through computational modeling. We also demonstrated that artesunate reduces cell viability, induced cytochrome c release, disrupts the mitochondrial membrane potential, and induces apoptosis when pediatric AML cells are treated with clinically relevant concentrations of artesunate. Lastly, the fact that methazolamide, an inhibitor of cytochrome c release, is antagonistic to artesunate’s anti-cancer activity shows that artesunate's activity is dependent on cytochrome c release. Taken together we used a multi-disciplinary approach to identify cytochrome c as a direct target of artesunate in pediatric AML cells.

## Methods

### Protein selection for modeling

Proteins interacting with heme and known to be present in the mitochondrion were collected from The Human Iron-Proteome project^40^. The structures of the proteins containing heme that were reported in this paper were collected from the Protein Data Bank (PDB)^41^. The PDB was searched for structures of the proteins that did not have a reported structure in the paper (because there is no experimental structure containing heme). First, the ID Mapping function on the Uniprot website was used to map IDs to Accession numbers in order to search the PDB. A total of 27 proteins are reported to interact with heme and localize in the mitochondrion. Of those, 13 have reported structures containing heme and structures were found for five more proteins in the PDB search. However, two of those structures were resolved using electron microscopy and were left out of this analysis. Two structures were selected for c-SRC, one of the active conformation and one of the inactive conformation.

For further investigation of cytochrome c, PDB files of the reduced (PDB code 2N9I) and oxidized (PDB code 2N9J) human protein were used for a more detailed docking study.

### Protein preparation and docking

Each PDB file was opened in the Molecular Operating Environment software (MOE—version 2020.0901) with biomolecular assembly selected. The protein was then prepared using the QuickPrep default settings. Then the Site Finder function was used to predict binding sites. Dummy atoms were created for the top predicted binding site. If there were additional binding sites that also scored very well, more than one was saved. Docking was performed in MOE using the following settings: Receptor: Receptor Atoms; Site: Dummy Atoms; Ligand: SDF file of artesunate downloaded from PubChem (CID 5,464,098); and default values for the method and score were used.

Then PDB files were saved for each protein containing the receptor atoms only and if heme is present, another PDB file is saved with the heme attached. A PDB file was saved for all the dummy atom groups representing different binding sites. A custom python and tcl script for Virtual Molecular Dynamics (VMD)^42^ was used to calculate the dimensions of docking boxes that contain the dummy atoms to be used with VinaMPI^43^. The receptor PDB files were prepared for docking using AutodockTools scripts and artesunate was prepared using Obabel. Docking was performed using VinaMPI on the University of Kentucky cluster, DLX. Additional docking studies were performed in MOE.

### UV–visible spectroscopy

Cytochrome c was ordered from Sigma-Aldrich. Oxidized cytochrome c was prepared at a 50.4 µM concentration in 50 mM PBS buffer at pH 7.4. Reduced cytochrome c samples was prepared at a 50.4 µM concentration in 50 mM PBS buffer at pH 7.4 under mild reducing conditions with DTT. UV–visible absorption spectra were obtained for both oxidized and reduced cytochrome c samples with and without 58.0 µM artesunate on a Thermo Scientific Nano Drop 2000 using a 1 mm path length. Effects on artesunate on the heme moiety were monitored at the Soret band at 409 nm and the Q band at 530 nm for the oxidized structure. For the reduced structure, the heme moiety was monitored by the red shifted Soret band at 415 nm, and the Q bands at 520 nm and 550 nm. Effects of artesunate binding and interaction with cytochrome c protein moieties were monitored at spectral regions consistent with the amide backbone absorbance at 206–220 nm and aromatic residues at 257–280 nm. Spectra were analyzed in Igor Pro 9 (WaveMetrics, Inc.). Difference Spectra were generated by subtracting protein spectra from the spectra obtained of the protein in the presence of artesunate for oxidized and reduced cytochrome c specimens, respectively.

### Cell lines and reagents

MV4-11 and THP-1 pediatric AML cells were purchased directly from ATCC. Passage number was monitored, and cells were refreshed using low passage number aliquots to ensure experiments were conducted in cell lines with similar passage numbers. MV4-11 cells were grown in Iscove’s Modified Dulbecco’s Medium (ATCC) supplemented with 10% Fetal Bovine Serum (FBS—Sigma Aldrich) and Penicillin/Streptomycin (Gibco); while THP-1 cells were grown in RPMI 1640 (ATCC) supplemented with 10% FBS, Penicillin/Streptomycin, and 0.05 mM 2-mercaptoethanol (MP Biologics). Cells were maintained in a 37 °C humidified incubator with 5% CO_2_. Purified artesunate (art), Carbonyl cyanide 3-chlorophenylhydrazone (CCCP) and methazolamide were purchased from MedChem Express.

### Isolation of cytoplasm and mitochondrial fractions

3 × 10^7^ MV4-11 or THP-1 cells were seeded into suspension culture flasks for each treatment group (vehicle control, 1 µM artesunate and 10 µM artesunate). Cells were treated for 24 h at 37 °C with 5% CO_2_, prior to isolation for both cytoplasmic and mitochondrial fractions using the Mitochondrial Isolation Kit (Sigma Aldrich, cat# MITOISO2). Following treatment cells were washed in PBS, the cell pellet was resuspended in 1.8 mL extraction buffer A, and cells were incubated on ice for 15 min. Next cells were homogenized using 30 strokes with a 2 mL Dounce homogenizer using the tight pestle (Sigma-Aldrich). Cell debris was removed by centrifugation at 600 × g for 10 min at 4ºC. The supernatant was transferred to a new microcentrifuge tube (VWR) and centrifuged at 11,000 × g for 10 min at 4 °C to separate the cytoplasm from the mitochondrial pellet. The supernatant was transferred to a new microcentrifuge tube labeled as the cytoplasmic fraction and was stored at − 20 °C for use in cytochrome c ELISA assays (details below). The pellet which contains intact mitochondria was resuspended in 200µL 1 × storage buffer and kept on ice for immediate use in cytochrome c oxidase activity assays (details below).

### Cytochrome c oxidase activity assay

A cytochrome c oxidase activity assay kit (Abcam) was utilized to determine if artesunate treatment effects the activity of cytochrome c oxidase (complex IV). Mitochondria were isolated (see above) from MV4-11 and THP-1 cells treated for 24 h with vehicle control, 1 µM artesunate, or 10 µM artesunate and incubated with reduced cytochrome c in a 96-well plate. The change in absorbance at 550 nm was measured every 30 s for 1 h using the kinetic loop parameters on a Varioskan LUX plate reader. Cytochrome c oxidase activity (units/1 × 10^6^ cells) was determined using the following equation cytochrome c oxidase activity = (ΔOD/Δt)/(E*cell number) where ΔOD is the difference in Abs_550nm_ between time_1_ and time_2_, Δt is the difference in minutes between time_1_ and time_2_, E is the molar extinction coefficient of reduced cytochrome c at 550 nm which is 7.04 for the components provided in the kit, and cell number is the number of cells in the mitochondrial isolation. Data is presented as the mean cytochrome c oxidase activity (units/1 × 10^6^ cells) from three independent experiments and statistical significance was determined by One-way ANOVA for each cell line followed by a Newman-Keuls multiple comparison test.

### Dose response assay

MV4-11 and THP-1 cells were seeded into white-walled 96 well plates at a density of 5000 cells per well in 50µL complete growth media. Next 12 drug stocks were made by serially diluting 200 mM stocks of artesunate or methazolamide 1:3 in 100% DMSO; subsequently, each stock was diluted 1:500 in complete media to generate 12 drug dilutions at twice the desired final concentration. Lastly, 50µL of each 2 × drug dilution was added per well containing 50 µl of cells so the final volume in each well was 100µL and the final DMSO concentration was 0.1% in each well. Each drug concentration was tested in duplicate wells, while triplicate wells were treated with 0.1% DMSO (vehicle) as an untreated control. Cells were incubated with drugs for 96 h prior to assessing cell viability using CellTiter-Glo 2.0 (Promega) viability assay. Data is presented as the % viability of treated cells normalized to 0.1% DMSO treated control cells and non-linear regression dose response curves using a four-parameter log-logistic model were fit to the data from at least three independent experiments using GraphPad Prism (version 5.01).

### Synergy

To assess drug interactions between artesunate and methazolamide drug response assays were preformed similarly to the method above; however, a 6 × 6 matrix design was used to assay pairs of drugs alone and in combination with five serially diluted concentrations of each drug. Cell viability was assessed following a 96 h treatment using CellTiter-Glo 2.0. Each well was normalized to untreated control cells which were grown in media with 0.2% DMSO and the percentage of viable cells was determined. R statistical software, specifically the snergyfinder package (version 1.10.4)^44^, was used to generate a synergy score using the Bliss independence model^27^ and the Loewe additivity model^28,29^.

### C*ytochrome c ELISA*

Cytoplasmic fractions of MV4-11 and THP-1 cells treated for 24 h with vehicle control, 1 µM artesunate, or 10 µM artesunate were analyzed for cytochrome c levels using the Quantikine ELISA Human Cytochrome c Immunoassay (R&D Systems) following manufacture instructions. Cytoplasmic fractions were diluted 1:5 in enzyme dilution buffer and 100µL diluted sample or cytochrome c standard was loaded to duplicate wells of a 96-well plate pre-coated with anti-cytochrome c antibodies and containing 100µL calibrator diluent RD5P from the ELISA kit. The plates were incubated for 2 h at room temperature and then washed 4 times with ELISA wash buffer. Each well was then incubated for 2 h with human cytochrome c conjugate before washing and incubation for 30 min with a substrate solution. After 30 min stop solution was added and the absorbance at 450 nm (background control) and 540 nm were read on a Varioskan LUX (ThermoFisher) plate reader. The corrected absorbance was determined by subtracting the Abs_450nm_ from the Abs_540nm_ and the amount (ng/mL) of cytochrome c in each sample was determined using the equation of a linear regression line generated by standard curve generated by serial dilutions of human cytochrome c standard provided in the ELISA kit. The data was normalized the amount of protein in each sample by dividing the ng/mL cytochrome c in each well by the amount of protein (µg/mL) in each well as determined by a BCA protein assay (Promega). Data is presented as the fold change in cytochrome c (ng cytochrome c/µg total protein) in artesunate treated cells normalized to matched vehicle control cells from at least three independent experiments. A One-way ANOVA followed by a Tukey’s Multiple Comparison Test was performed for each cell lines using GraphPad Prism (version 5.01) to determine if artesunate treatment resulted in a statistically significant change in cytoplasmic cytochrome c levels.

### MitoTracker and Caspase 3/7 staining

MV4-11 and THP-1 cells were seeded at a density of 2 × 10^5^ cells/mL in media containing 0.1% DMSO, 1 µM artesunate, 10 µM artesunate, or 40 µM CCCP and were incubated at 37ºC with 5% CO2 for 48 h. After treatment 500µL of cells were centrifuged at 500×g for 5 min, the cell pellet was resuspended in 100µL complete growth media with 5 µg/mL Hoechst 33,342 trihydrochloride trihydrate (Invitrogen) and either 25 nM MitoTracker Orange CMTMRos (ThermoFisher) or 5 µM CellEvent Caspase-3/7 green detection reagent (ThermoFisher) and incubated at 37ºC with 5% CO_2_ for 30 min. Cells were washed with phosphate buffered saline (PBS—Sigma Aldrich) containing 5% FBS and then fixed in 4% paraformaldehyde for 15 min at room temperature. After fixation cells were washed and resuspended in PBS with 5% FBS and 100µL stained cells were loaded into triplicate wells of a black-walled µClear 96-well plates (ThermoScientific). Cells were imaged using the CellInsight C × 7 High Content Analysis Platform (ThermoScientific) and quantification of MitoTracker orange signal or Caspase-3/7 green detection signal was performed using the HCS Studio software (ThermoScientific). MitoTracker orange signal was normalized to vehicle treated control cells and was expressed as the percent maximum (control) signal + / − SD. Caspase-3/7 activity signal was normalized to vehicle treated control cells and expressed as the fold increase over control + / − SD. Statistical analysis was performed using GraphPad Prism (version 5.01).

### Statistical analysis

To assess if the difference in artesunate IC50 values were statistically significant, the IC50 values calculated from multiple independent experiments along with the standard error for each experiment was graphed using GraphPad Prism 5. A two-tailed t-test was used to determine if the mean IC50 between two different cell lines. *P*-values are reported in the figure legends and results with a *P*-value less than 0.05 indicating a statistically significant difference. Differences in MitoTracker and Caspase-3/7 staining were determined for each cell line using a Two-way ANOVA followed by a Dunnett’s multiple comparison test to compare each treatment to 0.1% DMSO treated control cells. Differences in cytoplasmic cytochrome c and cytochrome c oxidase activity were determined using a One-way ANOVA for each cell line followed by a Tukey’s or Newman-Keuls Multiple comparison test respectively.

### Supplementary Information

Supplementary Information.

## Data Availability

Data for the docking analysis was gathered from the Protein Data Bank (https://www.rcsb.org/) and PubChem (https://pubchem.ncbi.nlm.nih.gov/). Docking result files can be made available upon request.
