# Supplementary material for: Artesunate acts through cytochrome c to inhibit growth of pediatric AML cells
Source: Sci Rep. 2023 Dec 16;13:22383. doi: 10.1038/s41598-023-49928-y (PMC10725448; doi:10.1038/s41598-023-49928-y)
Supplement: Supplementary file 1 — Supplementary Information. [file 41598_2023_49928_MOESM1_ESM.pdf]

Supplemental Table 1: Results of computational modeling of artesunate binding with mitochondrial heme containing proteins ranked based on MOE score

PDB code	Uniprot ID	Protein name	Protien function[1]	MOE
1BE3	CYB_HUMAN	cytochorme b	a subunit of complex III in the electron transport chain	-9.8015404
3PM0	CP1B1_ HUMAN	cytochorme P450 1B1	metabolism of aromativ hydrocarbons and 17beta-estradiol	-9.3473206
4ZGX	C11B2_ HUMAN	cytochrome P450 11B2	steroid biosynthesis	-8.7155085
4D6U (92)	CY1_ HUMAN	cytochorme c1	a subunit of cytochorme bc1 complex which is part of complex III in the electron transport chain	-8.5261269
4YTP (91)	C560_ HUMAN	succinate dehydrogenase complex subunit c	one of core subunits of complex II in the electron transport chain	-7.9289389
3K9V (84)	CP24A_ HUMAN	cytochrome P450 24A1	regulation of vitamin D3 and calcium homeostasis	-7.3895473
1OCC (91)	COX1_ HUMAN	cytochorme c oxidase 1	oxidizes cytochorome c as part of complex IV in the electron transport chain	-7.3139892
3N9Y	CP11A_ HUMAN	cytochome P450 11A1	steroid hormone biosythesis	-7.0265775
3NER	CYB5B_ HUMAN	cytochrome B5 type B	nitritic oxide biosythesis pathway	-6.8957138
3QM4 (90)	CP2D6_ HUMAN	cytochrome P450 2D6	oxidative metabolism of drugs	-6.6209002
3W1W	HEMH_ HUMAN	ferrochelatase	heme synthesis	-6.5933437
3NH6	ABCB6_ HUMAN	ATP-binding cassette trhansporter ABCB6	ATP-dependent importer of porphyrins into the mitochondria	-6.0825801
1SOX (68)	SUOX_ HUMAN	sulfite oxidase	catalyzes the oxidation of sulfite to sulfate in the oxidative degradation of cysteine and methionine	-5.9675045
2SRC/1Y57	SRC_ HUMAN	SRC1	non-receptor tyrosine protein kinase	-5.9594793
1J3S	CYC_ HUMAN	cytochorme c	shuttle between complex III and complex IV in electron transport chain, regulation of apoptosis	-5.7792711
3R8J	HEBP2_ HUMAN	heme binding protein 2	enhances mitochondrial membrane permeability, role in loss of mitochondrial memebrane potential	-5.778965

[1] functional information from genecards.org

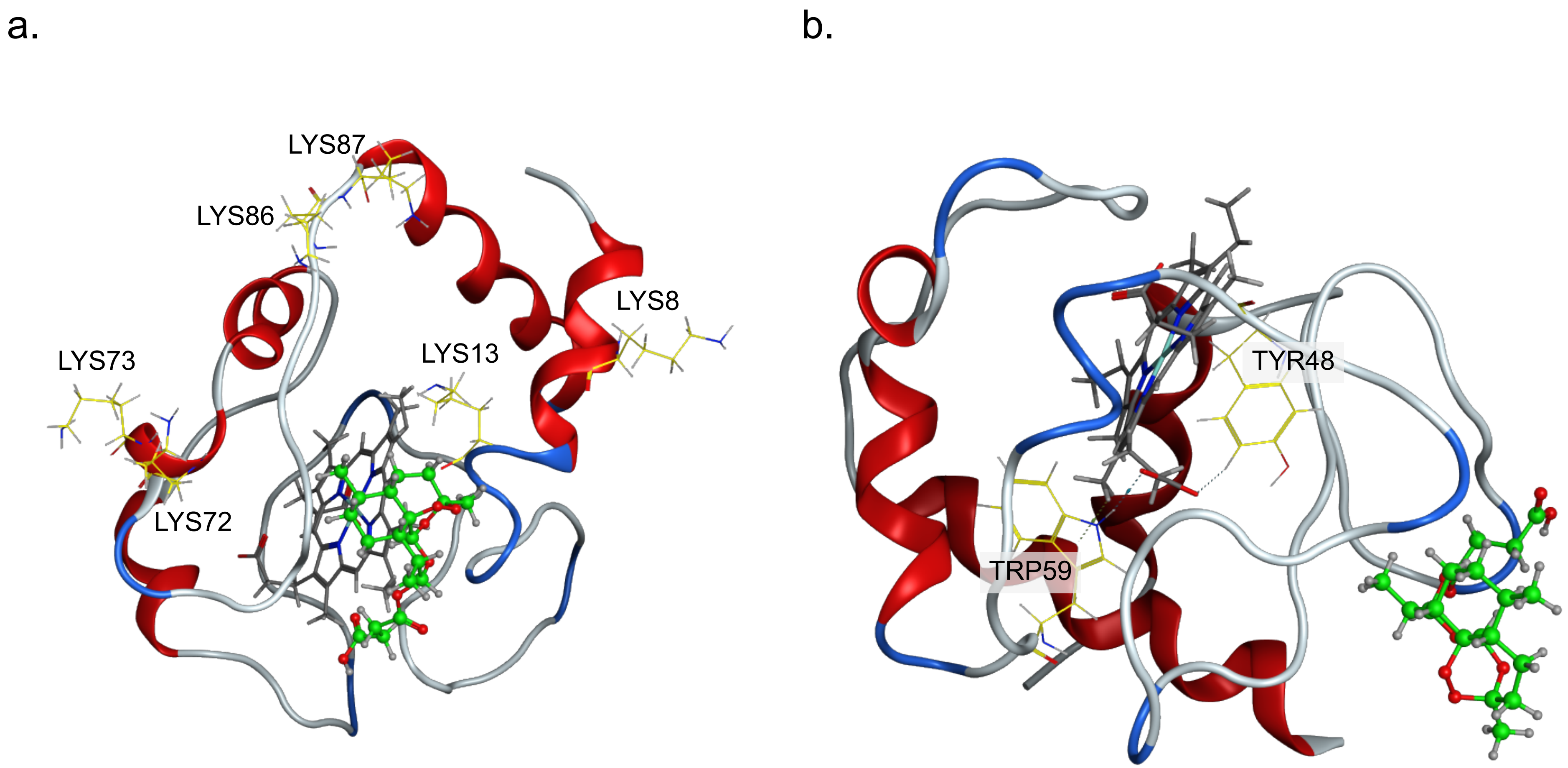

Supplemental Figure 1: Artesunate in top ranked binding site of reduced (a) and oxidized (b) cytochrome c as determined by MOE with key a mino acids labeled. a) Artesunate binds to reduced cytochrome c in close proximity to 6 key lysine (LYS8, 13, 72, 73, 86, 87) residues involved in binding of reduced cytochrome c to cytochrome c oxidase. b) Artesunate is predicted to bind oxidized cytochrome c within 15 angstrom of t ryptophan 59 (TRP59) and tyrosine 48 (TYR48) both of which have roles in stabilizing the heme and regulating cytochrome c induction of apoptosis. Artesunate (green), heme group (gray), key amino acids (yellow).

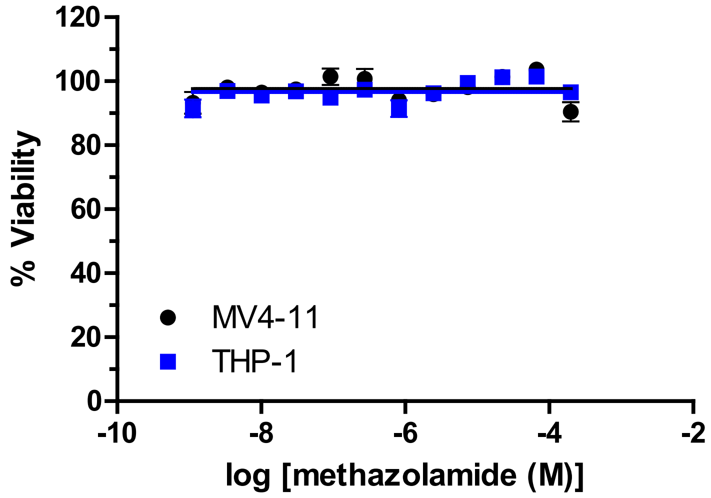

Supplemental Figure 2: MV4-11 and THP-1 cells are not sensitive to methazolamide alone. MV4-11 (black) and THP-1 (blue) cells were treated with increasing concentrations of methazolamide for 96hr prior to assess its effect on cell viability. Data is expressed as the mean +/- SD % viability normalized to matched 0.1% DMSO control treated cells from 4 independent experiments.
